# Best practices for clinical trials data harmonization and sharing on NHLBI bioData catalyst (BDC) learned from CONNECTS network COVID-19 studies

**DOI:** 10.1017/cts.2025.52

**Published:** 2025-03-26

**Authors:** Jeran K. Stratford, Huaqin Helen Pan, Alex Mainor, Edvin Music, Joshua Froess, Alex C. Cheng, Alexandra Weissman, David T. Huang, Elizabeth C. Oelsner, Sonia M. Thomas

**Affiliations:** 1 RTI International, Research Triangle Park, NC, USA; 2 Vanderbilt University Medical Center. Nashville, TN, USA; 3 Department of Epidemiology, University of Pittsburgh School of Public Health, Pittsburgh, PA, USA; 4 Department of Emergency Medicine, University of Pittsburgh School of Medicine, Pittsburgh, PA, USA; 5 Department of Critical Care Medicine, University of Pittsburgh School of Medicine, Pittsburgh, PA, USA; 6 Division of General Medicine, Columbia University Irving Medical Center, New York, NY, USA

**Keywords:** Data harmonization, BioData Catalyst, clinical trials, COVID-19

## Abstract

The need for collaborative and transparent sharing of COVID-19 clinical trial and large-scale observational study data to accelerate scientific discovery and inform clinical practice is critical. Responsible data-sharing requires addressing challenges associated with data privacy and confidentiality, data linkage, data quality, variable harmonization, data formats, and comprehensive metadata documentation to produce a high-quality, contextually rich, findable, accessible, interoperable, and reusable (FAIR) dataset. This communication explores the experiences and lessons learned from sharing National Heart Lung and Blood Institute (NHLBI) COVID-19 clinical trial (including adaptive platform trials) and cohort study datasets through the NHLBI BioData Catalyst® (BDC) ecosystem, focusing on the challenges and successes of harmonizing these datasets for broader research use. Our findings highlight the importance of establishing standardized data formats, adopting common data elements and creating and maintaining robust data governance structures that address common challenges (i.e., data privacy and data-sharing limitations resulting from informed consent). These efforts resulted in a set of comprehensive and interoperable datasets from 5 clinical trials and 13 cohort studies that will enable downstream reuse in analyses and collaborations. The principles and strategies outlined, derived through experience with consortia data, can lay the groundwork for advancing collaborative and efficient data sharing.

## Introduction

The rapid onset of the COVID-19 pandemic resulted in a situation requiring unprecedented speed and collaboration to understand the effectiveness of potential therapies. In response, the National Heart, Lung, and Blood Institute (NHLBI) initiated the Collaborating Network of Networks for Evaluating COVID-19 and Therapeutic Strategies (CONNECTS) program, a component of the Accelerating COVID-19 Therapeutic Interventions and Vaccines (ACTIV) public–private partnership to develop a coordinated research strategy for prioritizing and accelerating the development of the most-promising treatments and vaccines [1]. In addition to these five clinical studies, NHLBI funded a large Collaborative Cohort of Cohorts for COVID-19 Research (C4R) study from 14 ongoing NHLBI cohorts with rich pre-COVID data [2].

Clinical study data represent a substantial investment in time, money, and energy. Publishing the primary and secondary objectives of the study in a scientific journal is one mechanism to realize the potential of these data; however, maximizing the value of trial data requires responsible and ethical data sharing. Sharing scientific data accelerates discovery by enabling validation of results, providing access to high-value datasets, and promoting data reuse for future studies. Appropriate data sharing requires intentional effort to provide high-quality data with sufficient supporting information, including clear documentation of assumptions, data collection design choices, caveats to data combination, and limitations arising from consent restrictions, enabling others to understand and appropriately reuse the data.

To advance data sharing, the National Institutes of Health (NIH) issued the Data Management and Sharing (DMS) policy (NOT-OD-21-013), requiring submission and compliance with a DMS plan for scientific data [3]. Although CONNECTS predates this policy, NHLBI requires DMS responsibilities for all CONNECTS projects. Aligned with the DMS policy and Findability, Accessibility, Interoperability, and Reuse (FAIR) principles [4], CONNECTS project teams prioritized data standardization and harmonization to the CONNECTS common data elements (CDEs) [5]. This approach, coupled with continuous publication of both raw data (collected source data) and harmonized data (variables mapped to CDEs), facilitated timely and accessible data sharing.

CONNECTS study data was deposited in the data repository of NHLBI BioData Catalyst® (BDC), a cloud-based ecosystem that offers researchers scientific data, analytic tools, applications, and workflows in secure workspaces [6]. Study data are supported by rich metadata, including key indices that enable effective dataset search and cohort building, and relevant context to promote appropriate interpretation of study data and results.

This communication focuses on the key considerations, lessons learned, and process of preparing and sharing high-quality FAIR data from CONNECTS clinical trials, associated mechanistic studies, and the C4R cohort of cohorts study on BDC. We share experiences in harmonizing and standardizing datasets, navigating consent challenges, preparing comprehensive data packages, balancing data sharing timelines and effort, sharing data from adaptive platform trials, and fostering collaboration for a multisystem submission. While specific needs may vary across studies, this communication summarizing the CONNECTS program can serve as a general guide for consortia-level data sharing, a reference for BDC submissions, and an example of successful DMS implementation.

### COVID-19 studies and programs

In response to the COVID-19 public health emergency, NHLBl funded five multisite clinical trials testing candidate host-tissue-directed interventions to reduce morbidity and mortality. More than 6,600 participants were enrolled from 2020 to 2023. Investigators evaluated 18 intervention strategies using 10 molecular agents across the care continuum (outpatient, inpatient, and post-discharge). The trials collected clinical observations and standardized patient-reported outcomes [7–16]. Two trials were adaptive platform designs in which new treatment arms were added as the study progressed. Furthermore, the C4R cohort studies systematically ascertained SARS-CoV-2 infections and outcomes for > 50,000 participants across 14 collaborating cohorts. These studies represent a rich source of pre-COVID-19 data collected over many years including sociodemographic, clinical, lifestyle data, and deep phenotyping (e.g., imaging, “Omics) [2]. Participants in these studies represent a diverse population from young adulthood to the elderly and reflect the racial/ethnic, socioeconomic, and geographic diversity of the United States. By leveraging these diverse datasets, researchers can gain a comprehensive understanding of the impact of COVID-19 across different populations, thereby obtaining generalizable and unbiased findings.

## Preparing data for sharing

Maximizing the value of CONNECTS clinical trial and C4R cohort data requires the application of the FAIR data principles. This complex, yet essential task requires addressing issues of variable standardization and harmonization, comprehensive metadata and supporting documentation, and data linkage, quality, formats, privacy, and confidentiality. Overcoming these issues ultimately produces a consistent and comparable dataset ready for sharing and analysis.

### Data standardization and harmonization plan

Standardization and harmonization promote the core FAIR principle of data interoperability, the ability to seamlessly exchange and integrate data across different systems and formats. Interoperability drives efficient data sharing, improves data quality, and enhances data analysis. A critical component of the CONNECTS trials was the development of CDEs, standardized concepts that precisely define the question being asked with a specified set of responses. CONNECTS CDEs developed by a multidisciplinary team including physicians, biostatisticians, informaticians, and trialists promote the standardized capture of essential data elements for COVID-19 research [5]. To facilitate implementation, the team created an implementation manual, REDCap case report forms (CRFs), and a Clinical Data Interchange Standards Consortium based data dictionary, available through the CONNECTS website [17]. NIH endorsed the CONNECTS organ support data elements, making them available through their CDE repository [18] for electronic data capture systems.

**Figure 1.**
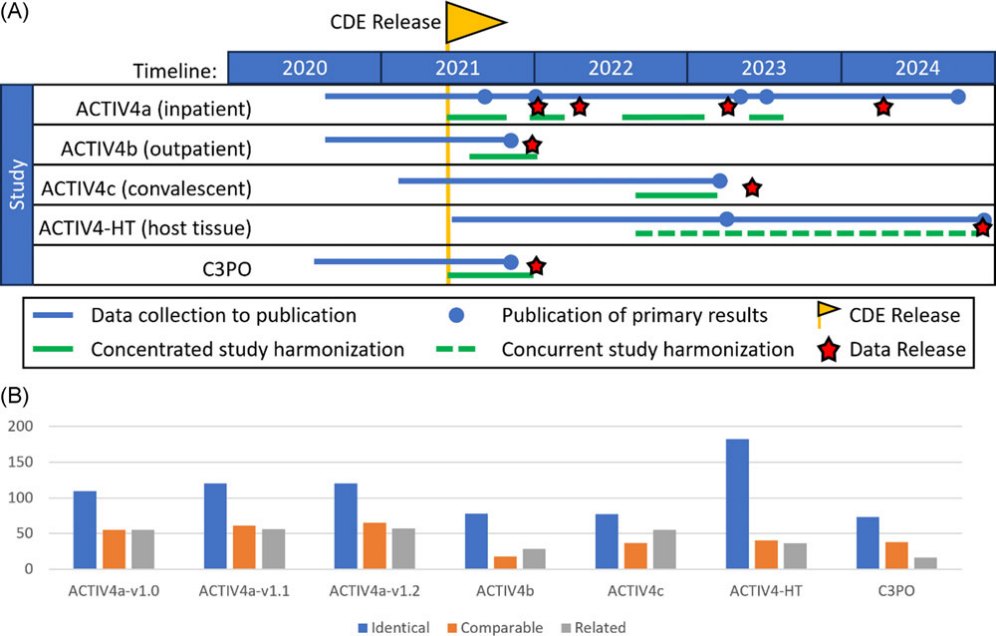
A. CONNECTS common data elements development and utilization. Many CONNECTS studies were ongoing (blue lines) prior to development and initial publication of the CONNECTS CDEs in June 2021 (yellow flag). Therefore, concentrated time for retrospective harmonization (solid green lines) was required to align study data with the CONNECTS CDEs to maximize dataset interoperability. In part, CDE adoption during study design coupled with concurrent data collection and intermittent harmonization (dashed green line) during ACTIV4-HT contributed to the reduction in time between study completion and dataset release (red stars). B. CONNECTS study variables mapped to CONNECTS CDEs. The count of mapping levels assigned to the study variable(s)/CDE pairing across CONNECTS studies was evaluated and visualized. An “Identical” mapping (blue) signifies study data was collected exactly as recommended by the NHLBI COVID-19 CDE. A “Comparable” mapping (orange) means that the study variable and NHLBI COVID-19 CDE are conceptually similar but differ in phrasing or response options. A “Related” mapping (gray) indicates that the study variable and the NHLBI COVDI-19 CDE covers a similar topic, but the mapping relationship is uncertain. ACTIV4-HT was the only study to adopt CONNECTS CDEs during study design, which greatly increased the number of “Identical” mappings, thus maximizing interoperability. Please note that ACTIV4a v1.0, v1.1, and v1.2 are different trial arms (drugs), not different versions of the same trial arm (drug).

While adoption of these CDEs during study design is now possible, the rapid deployment pace of CONNECTS trials prior to CDE publication necessitated retrospective harmonization of study data to the CDEs for the earliest studies. This labor-intensive process delayed data-sharing by 2–7 months, depending on the size and complexity of the study and the magnitude of the harmonization effort (Figure 1A). However, because up-front standardization was not an option, retrospective harmonization provided substantial value for subsequent users.

The heterogeneity of CONNECTS study designs and collection instruments created unique harmonization challenges. Study-specific strategies were developed using protocol-specific metadata describing collected variables from CRFs for the five trials: Clinical Trial of COVID-19 Convalescent Plasma in Outpatients (C3PO), ACTIV4a, ACTIV4b, ACTIV4c, and ACTIV4 Host Tissue (HT). A series of small mechanistic studies based on biospecimens collected in the ACTIV4a and ACTIV4-HT clinical trials each submitted laboratory assay data. The CONNECTS CDEs did not include these specialized assays.

The C4R cohort of cohorts approach enabled concurrent implementation of ancillary studies across the 14 cohorts, allowing for expedited study start-up. Cohort data coordinating centers (DCCs) collected data according to the established C4R protocol (including three waves of questionnaires [19], medical records for COVID-19 hospitalizations and deaths, and a dried blood spot serosurvey) and shared it with the C4R Data Coordination and Harmonization Center. Data were harmonized with the C4R data elements, but limited staff and budget precluded full harmonization with CONNECTS CDEs.

Mapping study variables to CDEs can introduce subjectivity and bias regarding content equivalence or mapping multiple variables to a single CDE. To mitigate bias and ensure accurate mapping, diverse perspectives in the mapping process are crucial. Harmonization of CONNECTS variables involved collaboration between the data managers and statisticians from the various study DCCs and CONNECTS administrative coordinating center (ACC) teams to create a harmonization template, guiding study teams in transforming variables mapped to the CDEs.

### Harmonizing clinical trial data

Upon receiving the completed protocol-specific harmonization template and guidance materials from the ACC harmonization team, the trial-specific data teams programmatically transformed the raw study data. Most studies implemented harmonization instructions using SAS. To enhance data accessibility, data were exported from SAS as comma-delimited text files, a widely accessible format for BDC submission.

Establishing content equivalence across studies was challenging due to differences in study designs (inpatient and outpatient), data collection methods (including evolving CRFs in adaptive platform trials), differences in data documentation format and level of detail, and inconsistent labeling of similar concepts. In some cases, mapping a study variable to a CDE and vice versa was not possible (e.g., incompatible collection scales or study-specific variables with no corresponding CDE), resulting in uneven adoption of CDEs across studies (Figure 1B), harmonized variables with missing values, and study variables unmapped to the CDEs and therefore not present in the harmonized dataset. To address this, both raw (study data as originally collected) and harmonized datasets were shared publicly. For maximal interoperability, we recommend that data requestors use the harmonized data whenever possible.

### Validation and quality of data harmonization

Following data transformation, the ACC harmonization team validated the data to ensure high fidelity. An R script programmatically evaluated each CDE domain, assessing the data structure and format (type, length), presence of required columns, adherence to controlled response options, missingness, and conditional field consistency. A *Pass* status was assigned to fields where all reported values follow the field definitions listed above. A field with any records that did not conform to all field definitions resulted in a *Fail*. A *Warning* status indicated that the data did not violate any field definitions but deviated from expectations and required human review. For example, warnings were issued for excessive missingness, numeric values outside of the expected range, or reported precision exceeding field definitions.

BDC was used for quality control (QC) evaluation for CONNECTS. Data were uploaded to a study-specific cloud-based project. Both study and curation teams could execute the R script from a BDC data studio and view the validation log generated for each CDE domain (e.g., vital signs, hospitalization) detailing the validation status (Pass/Fail/Warning) for each field with a description of any violation(s). Logs were consolidated into an Excel workbook for singular download. Validation issues prompted study teams to correct the data, upload the revised data, and re-validate.

This programmatic approach saved time and resources by efficiently identifying missing or incorrect values. Automated review took less than 10 minutes, enabling comprehensive QC, rather than spot checks of identified issues, confirming that fixes did not introduce new issues. The validation script is available on GitHub [20], enabling future studies to harmonize their own data with CONNECTS studies.

### Privacy and confidentiality

Dataset preparation also involved de-identification by removing sensitive information (i.e., personally identifiable information; protected health information) and any free text fields containing any sensitive information. Date variables were shifted by a consistent length of time (a random integer between 0 and 364 assigned at the participant level) from the true date, thus preserving the interval between dates. Study teams developed and shared documentation describing this method within the consortia to facilitate consistent implementation (Supplemental Information 1).

### Data linkage

The C4R studies include approximately 50,000 participants for 14 existing cohort studies (see Table 1). To integrate COVID-19 data with the pre-pandemic data, aligning C4R participant IDs with parent cohort IDs was crucial, enabling integrative analysis of the combined dataset. The CONNECTS ACC collaborated with investigators to document and link IDs during study registration, prior to data upload to BDC.

**Table 1.** Current data management and sharing status for CONNECTS studies. To request available study data sets, click the link in the “Data request” column at the study website https://nhlbi-connects.org/data-request

Study ID / Consortia	Study Name	Data Request Status	Comments
ACTIV4a	ACTIV4 ACUTE: Antithrombotics for Adults Hospitalized With COVID-19, Phase 1 - Heparin	Released	Three releases made data from this adaptive platform trial available as soon as possible. This study has 12-month follow-up data
	ACTIV4 ACUTE: Antithrombotics for Adults Hospitalized With COVID-19, Phase 2 - P2Y12 Inhibitors	Released	
	ACTIV4 ACUTE: Anti-thrombotics for Adults Hospitalized With COVID-19, Phase 3 - Crizanlizumab, SGLT2i, and some P2Y12 data	Released	
ACTIV4b	COVID-19 Positive Outpatient Thrombosis Prevention in Adults Aged 40-80	Released	One release for all study data
ACTIV4c	COVID-19 Post-hospital Thrombosis Prevention Study	Released	One release for all study data
ACTIV4 Host Tissue	NECTAR: Novel Experimental COVID Therapies Affecting Host Response	Released	One release for all study data
ACTIV4 Mechanistic Studies	Over eleven blood assay mechanistic studies from ACTIV4 Host Tissue patients that identify and quantify biomarkers of disease progress and response to treatment.	Released	Additional studies may be added over time.
C3PO	Clinical Trial of COVID-19 Convalescent Plasma in Outpatients	Released	Single release for all data
Collaborative Cohort of Cohorts for COVID-19 Research (C4R)	Atherosclerosis Risk in Communities (ARIC)	Released	14 cohorts, 13 with a single release of harmonized data
	Genetic Epidemiology of chronic obstructive pulmonary disease (COPD) Study (COPDGene)	Released	
	Framingham Heart Study (FHS)	Released	
	Northern Manhattan Study (NOMAS)	Released	
	Prevent Pulmonary Fibrosis (PrePF)	Released	
	REasons for Geographic and Racial Differences in Stroke (REGARDS)	Released	
	Severe Asthma Research Program (SARP)	Released	
	Coronary Artery Risk Development in Young Adults (CARDIA)	Expected Summer 2025	
	The Mediators of Atherosclerosis in South Asians Living in America (MASALA)	Released	
	Multi-Ethnic Study of Atherosclerosis (MESA)	Released	
	Hispanic Community Health Study/Study of Latinos (HCHS/SOL)	Released	
	Jackson Heart Study (JHS)	Released	
	Subpopulations and Intermediate Markers in COPD Study (SPIROMICS)	Released	
	Strong Heart Study (SHS)	Not submitting data	

### Supporting documentation and metadata

Each study provided a data dictionary detailing variables (description, label, length, type) for each dataset file and supplementary documentation (e.g., study protocol, survey instruments, CRFs, statistical analysis plan, de-identification readme), and additional documentation and methods needed for result reproducibility. Each research team created a master patient ID file to track protocol and consent versions where applicable. Teams combined supporting documentation with the raw and harmonized dataset to produce the data package.

For smaller mechanistic studies, the ACC harmonization team converted those data to CSV, ensured the data file contained the correct patient ID, and all data were de-identified and worked with the investigator in developing the corresponding data dictionary. For the C4R cohort studies, the C4R Data Coordination and Harmonization Center generated consistent documentation for all 14 cohorts, identified, and worked with the investigator in developing the corresponding data dictionary. For the C4R cohort studies, the C4R Data Coordination and Harmonization Center generated consistent documentation for all 14 cohorts.

## Sharing data

Data sharing requires careful planning and proactive consideration throughout the research lifecycle. By identifying and addressing data sharing requirements early, we can avoid delays and ensure data are readily available for dissemination at study conclusion. This approach aligns with the NIH’s emphasis on data-sharing and maximizes the impact of research findings.

### Repository selection

A crucial decision for data sharing is the selection of a suitable data repository(ies) with relevant security, retention, and access policies and search capabilities to make the data findable. Different repositories have varying requirements for data formats, documentation, metadata, data dictionaries, and how the data is shared. In CONNECTS studies, the selection of the NHLBI BioData Catalyst (BDC) platform was guided by funding opportunity specifications. Early identification of an appropriate data repository facilitated alignment of data management activities with repository-specific requirements to minimize rework as well as enabled concurrent effort towards other submission prerequisites, such as obtaining institutional certificates. Although this report focuses on our experience with the BDC platform, the lessons learned regarding data-sharing planning and implementation are broadly applicable to other data repositories and research projects.

### Managing data use limitation through consent

Sharing research participant data is intrinsically tied to consent. Participant consent delineates the terms under which data can be collected, shared, and used and serves as both a legal and ethical foundation for collaborative research. Different consent types, such as broad or specific consent, define the scope and limitations of data-sharing. Compliance with consent requirements is essential for respectful, responsible, and meaningful data sharing. CONNECTS trials had informed consent forms that clearly indicated participant consent included sharing of de-identified study data on BDC for use by other researchers. In addition, the CONNECTS C4R study used harmonized multidomain data from participants in long-term cohort studies (e.g., demographics, past medical history, neurocognitive testing, imaging, biomarkers) to examine factors that predict disease severity and long-term impacts of COVID-19 (Table 1). Preparation of C4R data for sharing revealed additional considerations due to variations in consent language from the parent cohort studies. During the review of the NIH Database of Genotypes and Phenotypes (dbGaP, a database of datasets [21]) registration, the cohorts worked with the Genomic Program Administrators (GPAs) to evaluate any differences in data use limitations (DUL) between C4R data and the parent study. If there were differences, a rigorous discussion was needed to balance sharing as broadly as possible with the consent obtained, often applying the more restrictive consent (e.g., disease specific compared to general research use) to the dataset. However, we recognize that some datasets could be split, and segments shared separately, each with their own appropriate DUL, allowing for a more-nuanced approach to data dissemination.

### Study registration

BDC manages access to the hosted controlled data using dbGaP’s data access approval mechanism [22]. Therefore, all datasets hosted in BDC require registration with dbGaP prior to upload [23]. Study teams provided study characteristics via the Data Submission Information (DSI) form and specific DUL in an Institutional Certification [24], which also assures NIH that the necessary infrastructure, policies, and procedures are in place for responsible and ethical data sharing consistent with applicable laws, regulations, and institutional policies. The timeline for obtaining an Institutional Certification typically takes several weeks to months and varies based on several factors, including study complexity, institutional experience, and involvement of an Institutional Review Board. CONNECTS studies initiated dbGaP registration concurrently with data collection and processing activities, a good lesson learned to avoid delays.

### Dataset submission and QC

**Figure 2.**
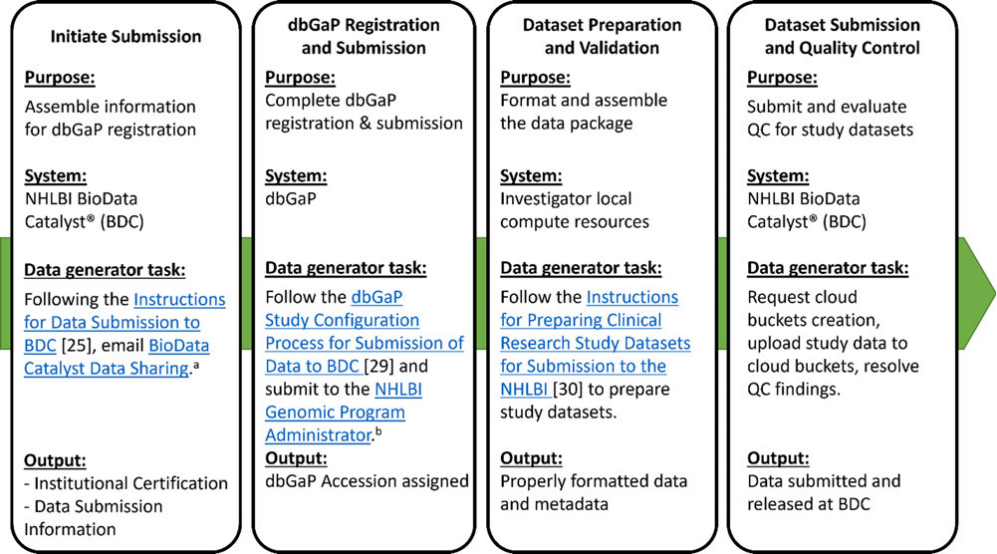
BDC submission workflow. Data generators who submitted datasets to BDC completed a multistep process involving multiple systems. The figure outlines tasks for this data generator led workflow for each step, with references to the relevant submission forms. The outcomes produced at each step that enable advancing to the next phase are outlined. dbGaP = database of genotypes and phenotypes; QC = quality control; BDC = NHLBI BioData Catalyst®; DMC = data management core; ^a.^ bdcatalystdatasharing@nih.gov, ^b.^ nhlbigeneticdata@nhlbi.nih.gov.

Data submission on BDC is a multistep process with tasks that data generators, or the study’s DCC, are responsible for at each step (Figure 2). In collaboration with BDC, the CONNECTS consortium became an early adopter of the ecosystem and tested the data-submission process for studies with non-genomic datasets enhancing its usability for future users. CONNECTS multiple data submissions provided test cases for optimizing the developed ingestion workflow, documentation, platform communications, and a “Frequently Asked Questions” resource covering topics, including the need for dbGaP registration to manage controlled access, ID masking, parent–child study registration, data use agreements and limitations, and submission links. Following study registration, study-specific cloud buckets were created on BDC for each DUL consent group indicated in the Institutional Certification form (e.g., general research use, health/medical/biomedical) for dataset upload and QC. Locally organizing data files by data- or consent-type facilitated easier upload of datasets to the correct consent-based cloud bucket. Quality assessment of uploaded CONNECTS data, completed by the BDC team, identified issues with subject consent mismatches, multiple ID variables, ID mismatch with parent studies, and the need for age/date de-identifications. Careful review of the “Instructions for Data Submission to BDC” [25], especially data preparation requirements [26], can minimize time and effort required for revision and resubmission.

### Adaptive trial release schedule, versioning, and updates

**Figure 3.**
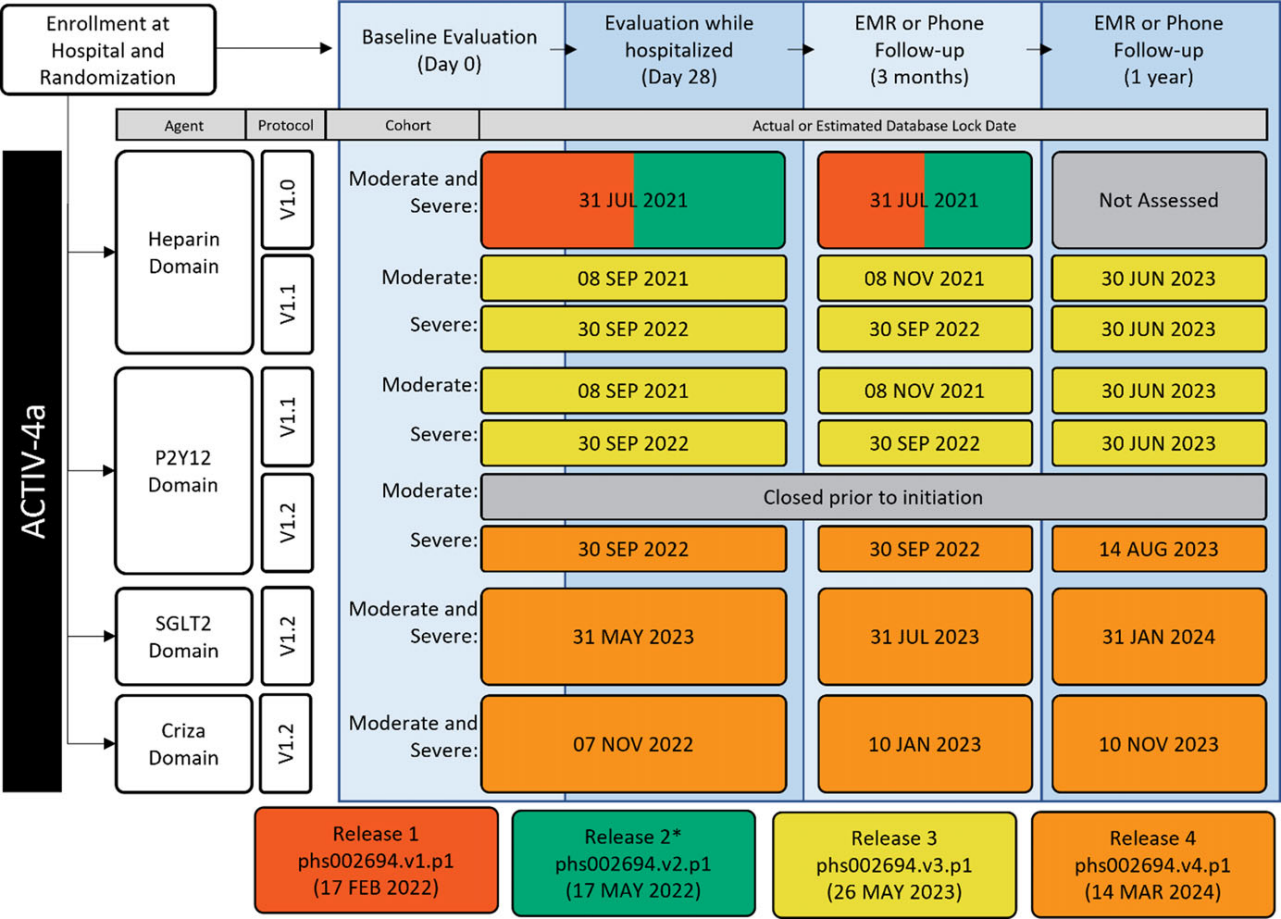
ACTIV4a adaptive platform trial data collection timelines. Adaptive platform trials allow for flexibility for interventions to enter or leave the platform based on a predefined decision algorithm. This flexibility results in staggered completion of longitudinal data collection (separate lock dates for each intervention). To make data available as soon as possible while balancing the effort required for data submission, harmonized datasets that are completed at the same time are aggregated (colors) into a single data release. One impact of this approach is the need to access multiple releases to obtain all data for one of the domains (P2Y12 for severe baseline disease). *Release 2 includes updated Release 1 data and is preferentially recommended for analysis. EMR = electronic medical records; SGLT2 = sodium-glucose cotransporter-2, criza = crizanlizumab.

ACTIV4a was an adaptive platform clinical trial. This dynamic and flexible design enabled modifications, including the addition and removal of treatment arms, while the trial continued. ACTIV4a assessed four commonly used treatments across two illness severity levels, with a 1-year post-acute follow-up. ACTIV4a prioritized sharing trial data with the research community shortly after each separate database lock with the hope of accelerating impactful pandemic research. However, the factorial design of overlapping treatments and staggered arm closures across the illness severity groups resulted in a near-constant cycle of database locks for specific study components. To efficiently balance harmonization and sharing effort with the desire for timely release, multiple study timepoint/components were aggregated for each release (Figure 3).

The complex study design necessitated careful communication explaining the differences between releases to avoid confusion. For instance, data in the first release needed some correction, resulting in a new version of the data and a second release. However, the ACTIV4a clinical trial had three protocol versions (v1.0, v1.1, v1.2) for four study drugs: Heparin, P2Y12 Inhibitors, Crizanlizumab, and SGLT2i. Datasets were named “v1.0,” “v1.1,” and “v1.2” corresponding to the three trial arms within the clinical protocol, not sequential versions of a single trial. This resulted in confusion regarding dataset content, as v1.1 in release 3 is an independent dataset, not an update of v1.0 that had previously resulted in release 2. Several discussions with the BDC support team were required to properly align the current data model. A diagram, such as that in Figure 3, is a helpful documentation and communication tool for adaptive trials for the study team and BDC staff, as well as future data users, to easily understand the contents of the various releases for the protocol.

ACTIV4-HT was also an adaptive platform trial. The trial began with four arms to assess two investigational new drugs (IND) versus placebo, but later added another IND treatment and matching placebo arm. The IND status of the active treatments and sharing of placebo patients across study arms, a feature often used in platform trial designs, spurred the decision to share data from all treatment arms in a single release.

From our experience, the timing of sharing data from arms in an adaptive platform trial varied based on the relative timing of database lock across arms, the speed of journal publication of each arm after submission, and design complications (shared placebo, factorial treatment designs) that impacted database lock. The push to share parts of ACTIV4a as soon as possible ultimately resulted in a more complex structure of public datasets than desirable, especially as retrospective review revealed no requests for the datasets within the first 3 years. In fact, early use of the ACTIV4a and ACTIV4-HT data resulted from direct collaboration between external researchers and the study team, such as joint meta-analyses with other trials [27–29], or sharing of biosamples and clinical data with approved mechanistic studies. Based on our experience, when platform trial database locks and corresponding primary publications occur within a short timeframe (e.g., less than a year), it may be more advantageous for adaptive platform trials to implement a sharing strategy like ACTIV4-HT with fewer releases that contain more comprehensive datasets than was implemented in ACTIV4a.

## Accessing CONNECTS data

All CONNECTS datasets for the five clinical trials, and 13 C4R cohort studies are expected to be available on BDC by summer 2025. The CONNECTS website provides study-specific links to initiate the data access request process [30], managed by dbGaP. Data availability, including for mechanistic studies, at the time of manuscript submission is provided in Table 1.

## Conclusions

The CONNECTS COVID-19 public use datasets from five clinical trials, mechanistic studies, and 13 C4R cohort studies are a valuable and comprehensive resource, offering a wealth of data regarding therapeutic options and outcomes for COVID-19. Although some challenges (e.g., data harmonization, QC) were similar across studies, each project encountered unique obstacles. The challenges to harmonize, document, and upload data were overcome by a proactive spirit of cross-team collaboration. Our experiences highlight the importance of early planning and incorporating data-sharing considerations into the initial study design, especially for adaptive platform trials, to limit potential rework and ease often encountered data-sharing pain points. Additionally, parallel execution of data-sharing activities and leveraging automated QC techniques can streamline the process and expedite timelines. Furthermore, harmonization of study data to CDEs enhances interoperability, enabling combining datasets across studies, which facilitates secondary analysis and fosters collaborative research to maximize the value of study data. By harmonizing and sharing these rich datasets on BDC, we not only provide a centralized hub for researchers to access and explore these analysis-ready datasets with accessible tools but also foster a culture of transparent and efficient data sharing.

## Supporting information

Stratford et al. supplementary material Stratford et al. supplementary material

## Supplementary material

The supplementary material for this article can be found at https://doi.org/10.1017/cts.2025.52.
