# Supplementary material for: Best practices for clinical trials data harmonization and sharing on NHLBI bioData catalyst (BDC) learned from CONNECTS network COVID-19 studies
Source: J Clin Transl Sci. 2025 Mar 26;9(1):e87. doi: 10.1017/cts.2025.52 (PMC12083401; doi:10.1017/cts.2025.52)
Supplement: Stratford et al. supplementary material [file S2059866125000524sup001.docx]

# Supplemental Information 1

## CONNECTS De-Identification Guidance

**Last updated: November 29, 2022**

As studies begin to prepare to upload data to BioData Catalyst, it is recommended that teams employ a uniform approach to de-identification to promote cross-study comparison to the greatest extent possible. **The study team is responsible for the de-identification of all data uploaded to BioData Catalyst in accordance with** [**NIH Data Sharing Standards**](https://osp.od.nih.gov/scientific-sharing/genomic-data-sharing/)**, as outlined in BioData** [**Catalyst Data Protection Guidance**](https://biodatacatalyst.nhlbi.nih.gov/data-protection/) **and** [**Data Generator Guidance**](https://bdcatalyst.gitbook.io/biodata-catalyst-documentation/data-management/biodata-catalyst-data-generator-guidance)**.** These standards apply to both raw and harmonized datasets uploaded by the study team, as well as any additional potentially identifiable data. The CONNECTS Data Standards Core is not responsible for ensuring data is properly de-identified in accordance with NIH Data Sharing Standards. The guidance below is not comprehensive. It is expected that this guidance will evolve as studies encounter new issues in the de-identification process.

### 1. Date De-identification

The CONNECTS Data Standards Core recommends date shifting instead of use of a reference date (e.g., days from randomization or consent) when de-identifying dates. Because the goal of CONNECTS is standardization across studies to the greatest extent possible, date shifting is preferred to reference dates to avoid any confusion in interpretation of Day 0 vs. Day 1 (and therefore all subsequent days) across studies. Additionally, not all studies under the CONNECTS umbrella include the same data collected in clinical trials (e.g., randomization date); use of date shifting instead of reference date will allow broad applicability and linkage to different types of studies in the future, including observational and non-randomized studies. This approach should be applied to both raw and harmonized datasets; dates across raw and harmonized datasets should be shifted by the same value.

Dates should be shifted by a consistent length of time for each record by a random integer from 0-364 days subtracted from the true date, thus preserving the interval between dates. For example, if a subject, Mary, had three sequential appointments with dates of April 2, April 15, and April 26, when the dates are shifted, each appointment will remain in order sequentially with the same interval between appointments November 16, November 29, and December 10. For dates where only a month and year is available, the day of the month should be imputed to the 15^th^ for date shifting purposes only. After a date using the 15^th^ of the month is created, the same date shifting method outlined above should be employed. The dummy day of month should then be returned to missing status and only the shifted month and year should be uploaded as the actual date. If only a year is available, no date shifting should occur, and day and month should be marked as missing.

For most domains (DM, MH, RSK, ORG, SYM, AE, CM, VS, LB, COVID, DS, INT, HO) follow the above guidance as illustrated in the examples below:

| **Date Elements Present (DD-MON-YEAR) * Indicates missing** | **Date Shifting Approach** |
| --- | --- |
| **-MON-YEAR | Impute DD as 15, date shift, return dummy DD to missing |
| **-***-YEAR | Do not date shift, retain YEAR as is |
| DD-***-YEAR | Remove DD, do not date shift, retain YEAR as is |
| **-MON-**** | Mark entire date as missing |
| DD-***-**** | Mark entire date as missing |
| DD-MON-**** | Mark entire date as missing |

For the VCC domain, capture the lowest level of granularity for dates common across **all** doses, to avoid potential confusion regarding the timing of multiple doses received. For example:

- - - If one dose has year+month, and the other has only year, drop month to only keep year for both, and do not date shift.
    - If one dose has year+month+day, and the other dose has only year+month, drop day to keep year+month for both, and date shift according to above guidance.
    - If one dose has year+month+day, and the other dose has only year, drop day+month to keep year for both, and do not date shift.

Date shifting leaves the project record intact and will not affect the actual saved dates in the project; merely alters the dates in their resulting format. If using REDCap, this function can be performed automatically upon data export.

### 2. Use of Free Text Fields

A select number of CONNECTS core CDEs require the input of free text fields. After harmonization review, studies should upload data for these fields only; any additional free text fields should be uploaded without data (but retaining the variable itself for matching to study CRFs). Study personnel must review data in this limited set of required free text fields to ensure no identifying data is included. Any free text data included in raw study data must also be reviewed by the study team to ensure proper de-identification.

### 3. Individuals 90 or Older

For individuals age 90 or above, ages should be aggregated into a single age grouping (“90”).
